# Investigation into the Importance of genes encoding ciliary proteins in congenital heart disease using whole exome sequencing

**DOI:** 10.1186/2046-2530-4-S1-P9

**Published:** 2015-07-13

**Authors:** V Hartill, C Logan, DA Parry, K Szymanska, K Ashcroft, K English, K Prescott, A Dobbie, S Barwick, C Bennett, J Goodship, E Sheridan, C Johnson

**Affiliations:** 1 Leeds Institute of Biomedical and Clinical Sciences, University of Leeds, Leeds, UK; 2 Department of Clinical Genetics, Leeds Teaching Hospitals NHS Trust, Leeds, UK; 3 Department of Congenital Cardiology, Leeds Teaching Hospitals NHS Trust, Leeds, UK; 4 Institute of Human Genetics, Newcastle University, Newcastle Upon Tyne, UK

## 

Congenital Heart Disease (CHD) is the most common congenital defect. Many families with left-right laterality defects and complex CHD have an unknown genetic aetiology. Many ciliopathies, including Primary Ciliary Dyskinesia (PCD), are associated with intracardiac defects. The role of primary cilia in cardiac morphogenesis remains unknown, although cardiac cilia have roles that are distinct from the definition of laterality at the embryonic node. We hypothesise that defects in genes important in the assembly and function of cilia are responsible for some inherited forms of CHD.

This research project aims to recruit families with a recurrence of CHD and to perform Whole Exome Sequencing (WES) to identify putative pathogenic variants and to delineate novel genetic causes of CHD.

Twelve families have now been recruited and WES has been carried out in seven of these families using paired-end sequencing. Data analysis follows a standardised pipeline to call and then filter variants in order to assess their pathogenic potential. Variants are prioritized on the basis of known or suspected function of the encoded protein, and publicly-available RNA expression data.

Variant filtering has allowed the identification of a limited number of candidate variants in recruited families. Of particular interest, a likely causative homozygous variant within a PCD gene has been identified in two siblings affected with heterotaxy, thus confirming a link between ciliopathies and CHD. The function and interactions of identified genes will be assessed, using cellular techniques and animal models, to provide insights into the pathogenesis of CHD.

